# The influence of chemotherapy on adenosine-producing B cells in patients with head and neck squamous cell carcinoma

**DOI:** 10.18632/oncotarget.23533

**Published:** 2017-12-20

**Authors:** Andreas Ziebart, Ulrich Huber, Sandra Jeske, Simon Laban, Johannes Doescher, Thomas K. Hoffmann, Cornelia Brunner, Edwin K. Jackson, Patrick J. Schuler

**Affiliations:** ^1^ Department of Neurosurgery, University Hospital Mannheim, University of Heidelberg, Mannheim, Germany; ^2^ Department of Otolaryngology, Head and Neck Surgery, Ulm University Medical Center, Ulm, Germany; ^3^ Department of Pharmacology and Chemical Biology, University of Pittsburgh School of Medicine, Pittsburgh, Pennsylvania, USA

**Keywords:** regulatory B cells, adenosine, head and neck cancer, cisplatin, methotrexate

## Abstract

**Introduction:**

Head and neck squamous cell carcinoma (HNSCC) strongly suppresses the immune system, resulting in increased metastasis and recurrent disease. Chemotherapy is part of the multimodal treatment but may further immunosuppression. Recently, we demonstrated that regulatory B cells (Breg), defined as CD19^+^CD39^+^CD73^+^ B cells, play a significant role in the production of immunosuppressive, extracellular adenosine (ADO). Here, we tested the influence of chemotherapy on Breg function.

**Results:**

In HNSCC patients, Breg were diminished in absolute number and frequency after chemotherapy (paired samples). Chemotherapeutic drugs had variable effects; while platinum-based chemotherapy decreased the expression of CD39, methotrexate led to a functional increase in CD39 expression and increased production of immunosuppressive ADO. These findings were confirmed in a second patient cohort. Surface expression of CD39 correlated strongly with the production of ADO as measured by mass spectrometry.

**Conclusions:**

Platinum-based anti-tumor-therapy reduces the number of adenosine-producing B cells and, consequently, potential immunosuppression within the tumor environment. Breg function in terms of ADO production and their potential capacity to suppress CD4^+^ T cells are promoted by methotrexate treatment amplifying anti-inflammatory therapeutic effects. Our results add to the understanding of how chemotherapeutic drugs can influence the human immune system and may therefore help to orchestrate standard oncologic therapy with new immune modulating approaches.

**Methods:**

Mononuclear cells were collected prospectively from HNSCC patients before and after chemotherapy (*n* = 18), from healthy donors (*n* = 20), and an additional cohort sampled several months after chemotherapy (*n* = 14). Frequency, phenotype, and function of Breg were determined by multicolor flow cytometry, ATP luminescence assay as well as mass spectrometry measuring 5′-AMP, ADO, and inosine. Isolated B cells were incubated with chemotherapeutic drugs (cisplatin, methotrexate, paclitaxel, 5-fluorouracil) *in vitro* for functional studies.

## INTRODUCTION

### B cells in cancer

The role of tumor-infiltrating B lymphocytes (TIL-B) in solid cancers has long been strongly underestimated [1]. It has recently become clear, that the presence of TIL-B frequently correlates with a good prognosis in a variety of solid cancers, including breast, colon, and lung cancer as well as head and neck squamous cell carcinoma (HNSCC) [2–4]. Furthermore, it has been suggested that the relevance of TIL-B as a prognostic marker may exceed the role of CD8^+^ T lymphocytes, given that the interaction of both cell types is mandatory for a sufficient anti-tumor response [5]. Additionally, their controversial function includes a tumor-promoting role [6] and moreover, several murine tumor models implement a pro-tumor effect of TIL-B [7, 8]. In addition, B cells inhibit the anti-tumor effect of vaccination against murine malignant melanoma and they induce radio-resistance in murine prostate cancer [9, 10]. This effect may be partly explained by conversion and attraction of regulatory T cells (Treg) into the tumor micro-environment [1]. However, it also reiterates the difficulty in correlating murine models with the human system.

Nevertheless, it may be worthwhile to further subdivide the B cell population into immune competent B cells and regulatory B cells (Breg). Breg are commonly characterized by their potential to produce immune suppressive mediators, e.g. IL-10 and granzyme B [11, 12]. In humans, the presence of IL-10 producing regulatory TIL-B (TIL-Breg) is associated with a worse prognosis in HNSCC and gastric cancer [13, 14]. Similarly, the increased frequency of circulating Breg is correlated with advanced tumor stages in human hepatocellular carcinoma [15]. It has further been shown that TIL-Breg suppress the proliferation of CD4^+^ and CD8^+^ T cells as well as NK cells requiring a direct cell contact [16]. Additionally, IL-10 secreting TIL-Breg induce immunosuppressive regulatory T cells in breast cancer patients [17].

### B cells and adenosine

Our research team recently described a new group of Breg defined by their ability to produce immune suppressive adenosine (ADO) from exogenous ATP using ectonucleotidases CD39 and CD73 [18]. The co-expression of both enzymes is found in the majority (>65%) of all peripheral B cells in healthy donors. As we have previously shown, a variety of other immune cell populations also carry CD39 and/or CD73 on their surface, including regulatory T cells (Treg) [19], mesenchymal stem cells (MSC) [20], T helper cells [21] as well as non-cellular exosomes [22]. However, among the immune cells B lymphocytes display the highest CD39/CD73 expression and the largest potential for production of ADO [18]. In addition, several tumor cell lines have also been shown to express ectonucleotidases, contributing to ADO metabolism [23].

### Adenosine in cancer

Recently, the role of adenosine in the progression of solid cancers has been discovered and investigated in detail [24]. In the tumor micro-environment of HNSCC, Treg upregulate the expression of ectonucleotidases [25]. In contrast, MSC display a decreased production of ADO when isolated from HNSCC tissue and compared to MSC from autologous healthy tissue [20]. Tumor cells of HNSCC and prostate cancer are stimulated by ADO via ADO receptor (ADOR_A2_) *in vitro*, indicating a pro-tumorigenic effect of exogenous ADO [26, 27]. In humans, the ectonucleotidase CD73 is a strong and independent prognostic marker in a large number of ovarian cancer and prostate cancer patients and is correlated with poor outcome [28, 29]. Both the immune suppressive effect and tumor-promoting effect make ADO an interesting therapeutic target. The ADO pathway can be inhibited either by inhibition of the enzymes CD39 and CD73 or by the blockade of ADO-specific receptors. While the modulation of CD39 is still in preclinical testing, an antibody against CD73 has recently entered the clinical trials stage for solid tumors (phase I, NCT02503774). Interestingly, caffeine acts as a natural inhibitor of ADOR_A2a_ and reduced tumor growth in a murine 3-MCA and melanoma model [30]. Similarly, the blockade of ADOR_A2a_ increases the efficacy of an anti-PD1 therapy in a murine breast carcinoma model [31, 32]. Based on these promising findings, a selective ADOR_A2a_ inhibitor is currently being tested in a clinical trial for advanced non-small cell lung cancer (phase I, NCT02403193). In summary, inhibition of ADO receptors can have two beneficial effects for cancer patients: (I) improved functionality of immune cells, and (II) decreased proliferation of cancer cells, making this a promising treatment option.

Despite the importance of ADO in cancer progression, very little is known about the influence of cancer therapy on ADO-producing immune cell populations. It is now clear that the frequency of Treg is slightly increased in patients with HNSCC. However, after chemoradiotherapy (CRT) the frequency of peripheral CD39^+^ Treg increases dramatically due to their relative resistance to CRT as compared to CD4^+^ T helper cells [33]. This may contribute to increased ADO levels and further immune suppression resulting in recurrent disease after CRT. Similarly, treatment with the EGF inhibitor cetuximab increased the frequency of CD39^+^ Treg in the micro-environment of HNSCC [34]. In contrast, a decrease in frequency and number of CD39^+^ Treg was observed after vaccination with p53-specific dendritic cells with a possible immune enhancing effect due to decreased ADO concentrations [35].

In the present study we describe for the first time the influence of chemotherapy on adenosine-producing Breg in two independent patient cohorts with HNSCC.

## RESULTS

### Changes in frequency, absolute numbers and phenotype of B cells before and after CRT

**Figure 1:**
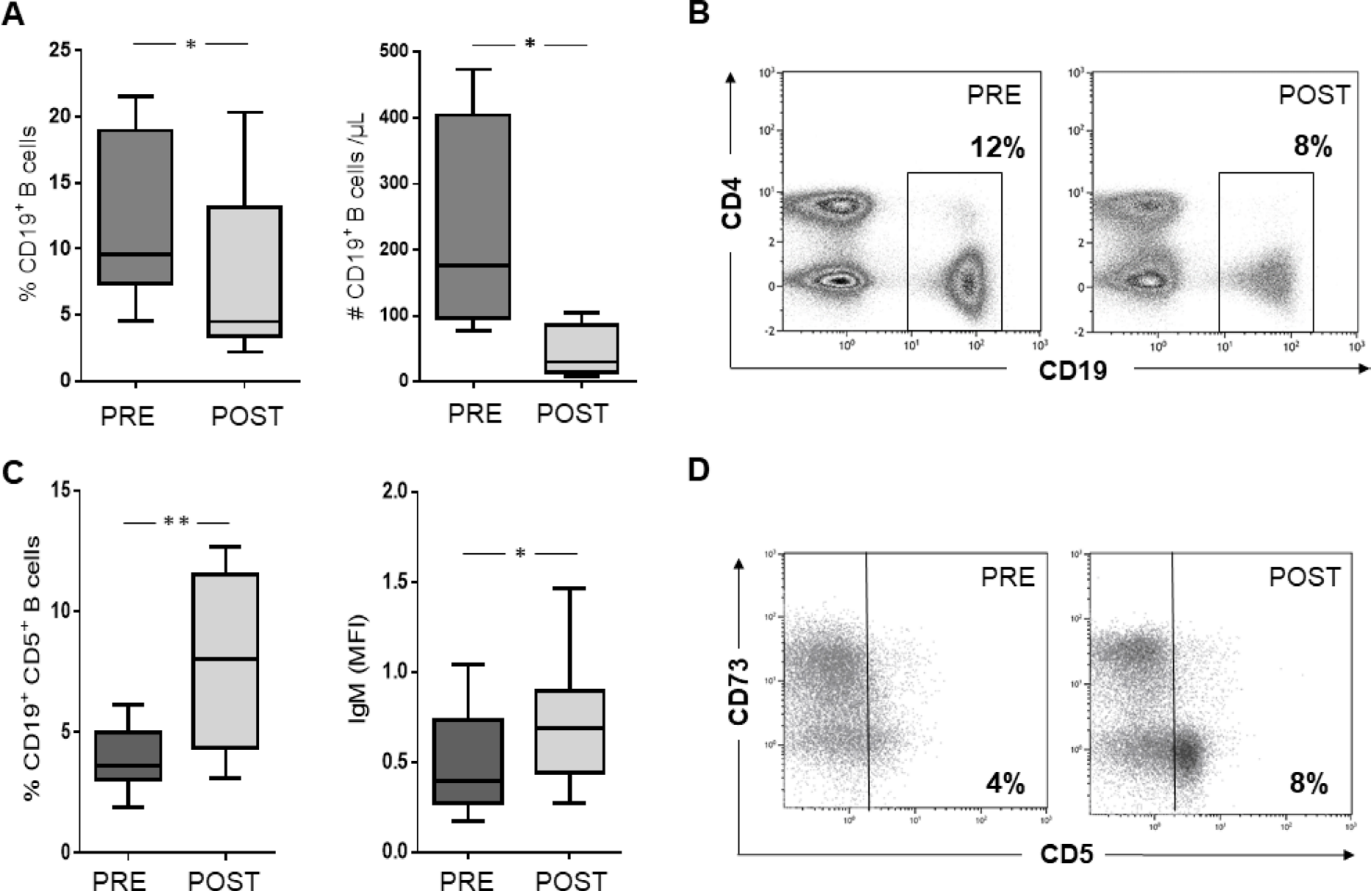
(**A**) and (**B**) The frequency (*n =* 15) and absolute number (*n =* 4) of B cells were significantly reduced in the peripheral blood of HNSCC patients after CRT as compared to pretreatment measurements. (**C**) CRT induced an increased expression of CD5 and IgM within the B cell compartment. (**D**) Density plot of one representative patient demonstrating an increasing portion of CD19+CD5+ B cells after CRT.

For each HNSCC patient of cohort #1, the frequency and absolute number of CD19^+^ B cells in the peripheral blood were compared before and after CRT. Both the frequency and the absolute number of CD19^+^ B cells were decreased (each *p <* 0.05) after CRT (Figure 1A). Representative density plots are shown in Figure 1B. In cohort #1, the frequency of CD4^+^ T cells also decreased significantly (Supplementary Figure 1A), while the frequency of CD8^+^ T cells was not significantly affected, confirming the data from previous publications [33]. While these changes applied to patients treated with a platinum-based chemotherapy, patients treated with methotrexate showed no alterations (Supplementary Figure 1B).

Furthermore, B cells in patient cohort #1 were tested by flow cytometry for expression of various immunologic surface markers. IgM surface expression, as well as the IgM^+^ B cell subset, were significantly increased after CRT (Supplementary Figure 1C). In addition, there was an increase in the CD19^+^ CD5^+^ B cell compartment after CRT, which is considered critical regarding the promotion of further tumor growth (Figure 1C, 1D) [37]. Both surface markers, IgM and CD5, were found to be unchanged after methotrexate therapy. B cells were negative for CD26 and no expression was induced by CRT. Expression rates and percentages of CD25^+^, PD1^+^, CCR7^+^, IgA^+^, and CD40^+^ B cells also showed no significant alteration after treatment (Supplementary Figure 1E and 1F).

### Phenotypic characterization of ADO-producing B cells

**Figure 2:**
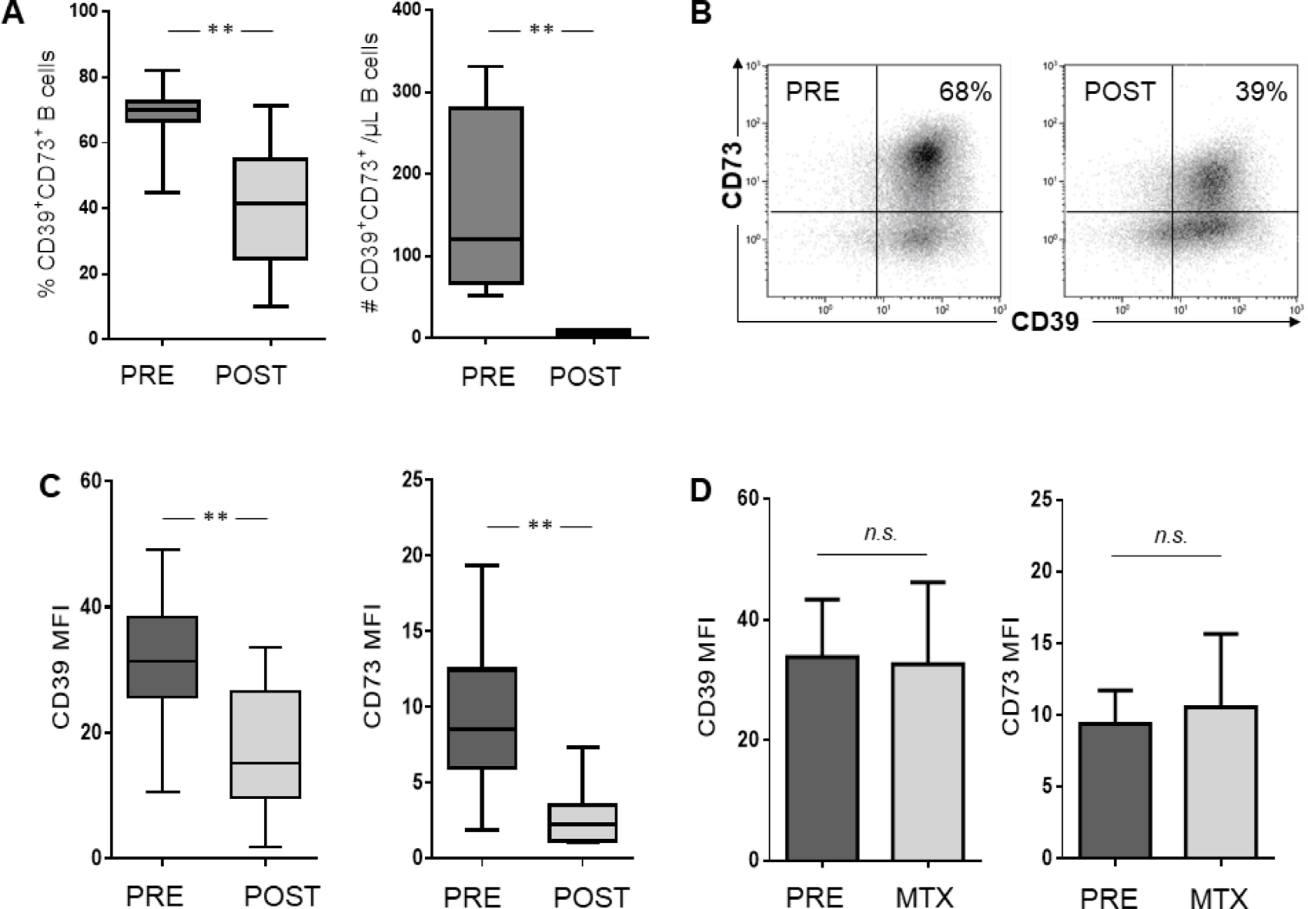
Phenotypic characterization of B cells in patients with HNSCC before and after treatment with CRT, respectively Isolated PBMC were stained for flow cytometry and examined for surface expression of ectonucleotidases CD39 and CD73. (**A**) Frequency (*n =* 15) and absolute number (*n =* 4) of adenosine producing B cells defined by the co-expression of CD39 and CD73. (**B**) Density plot of one representative subject showing CD39 and CD73 expression in CD19+ gated cells. Percentages of CD19+CD39+CD73+ cells are indicated in the relevant quadrant. Box plots showing reduced surface expression of CD39 and CD73 as mean fluorescence intensity (MFI) in patients treated with cisplatin/carboplatin (**C**) while bar charts before and after methotrexate treatment are show no significant difference (**D**).

In patient cohort #1, flow cytometry analysis showed that up to 82% of B cells co-expressed CD39 and CD73 on their cell surface. As previously reported, these cells demonstrate an immunosuppressive potential by hydrolyzing exogenous ATP to ADP, 5′-AMP, and ADO [18]. Therefore, we were especially interested in therapy-induced changes in this Breg subset. Within the CD19^+^ B cell compartment, the frequency and the absolute number of these CD39^+^CD73^+^ Breg was significantly decreased after CRT (*p <* 0.005) (Figure 2A, 2B). Consequently, the subsets of CD39^+^CD73^neg^ as well as CD39^neg^CD73^+^ B cells were increased (*p <* 0.01, data not shown). As shown in Figure 2C, the mean fluorescence intensity (MFI) of both ectonucleotidases, CD39 and CD73, was significantly reduced in the CD19^+^ B cell compartment after platinum-based chemotherapy (*p <* 0.001). Interestingly, MTX treatment showed no reduction in the ectonucleotidases (Figure 2D) and also no decrease in co-expressing cells (Supplementary Figure 1D).

### *In vitro* changes by cytostatic drugs

**Figure 3:**
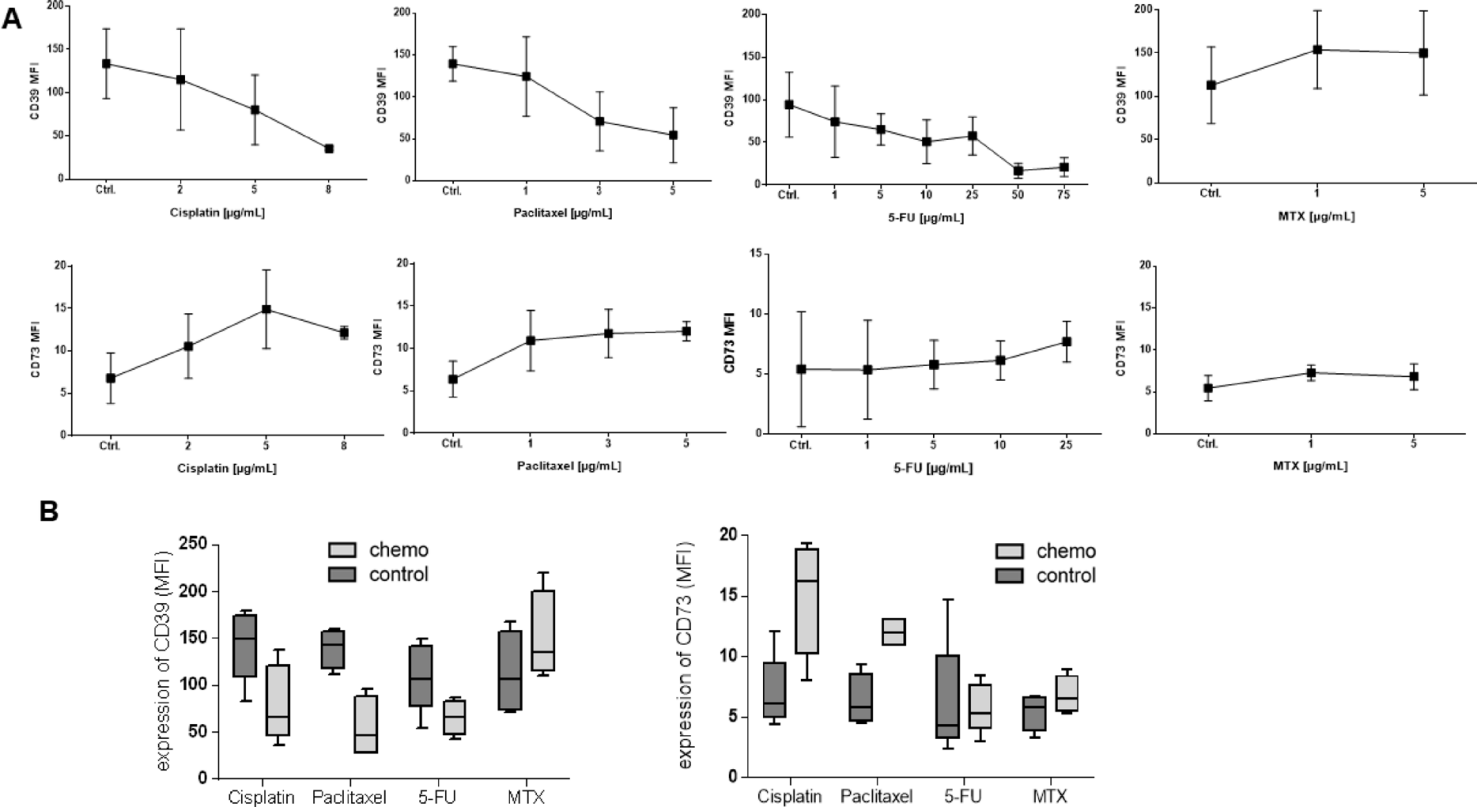
*In vitro* expression levels of CD39 and CD73 on B cells Human B cells (precultured with IL-4, CD40L, and hemagglutinin) from healthy donors were separated as described and cultured in the presence of therapeutic concentrations of cytostatic drugs in RPMI medium. (**A**) Data are means ± standard deviation determined for all CD19+ B cells obtained from at least 3 normal donors. (**B**) Box plots showing mean fluorescence intensity of CD39 and CD73 in B cells precultured with 5 µg/mL of one cytostatic drug, respectively.

To test the different effects of cytostatic drugs on ADO-producing B cells, isolated B cells of healthy donors were treated with chemotherapy *in vitro* for 7 days as described above. Cytostatic drugs were chosen from regimens used in standard therapy for HNSCC patients in clinically administered concentrations (cisplatin, paclitaxel, 5-FU and MTX). Cisplatin, paclitaxel, and 5-FU induced a dose-dependent decrease in CD39 surface expression (*p <* 0.05). However, methotrexate induced a significant increase in CD39 expression on B cells (*p <* 0.05, Figure 3A), while the mean fluorescence intensity of CD73 was significantly increased after incubation with cisplatin and paclitaxel (each *p <* 0.05). 5-FU and methotrexate did not influence surface expression of CD73 (Figure 3B). Cetuximab did not show either of these significant influences regarding the expression of CD39 and CD73 (data not shown).

### ATP hydrolysis by luminescence

**Figure 4:**
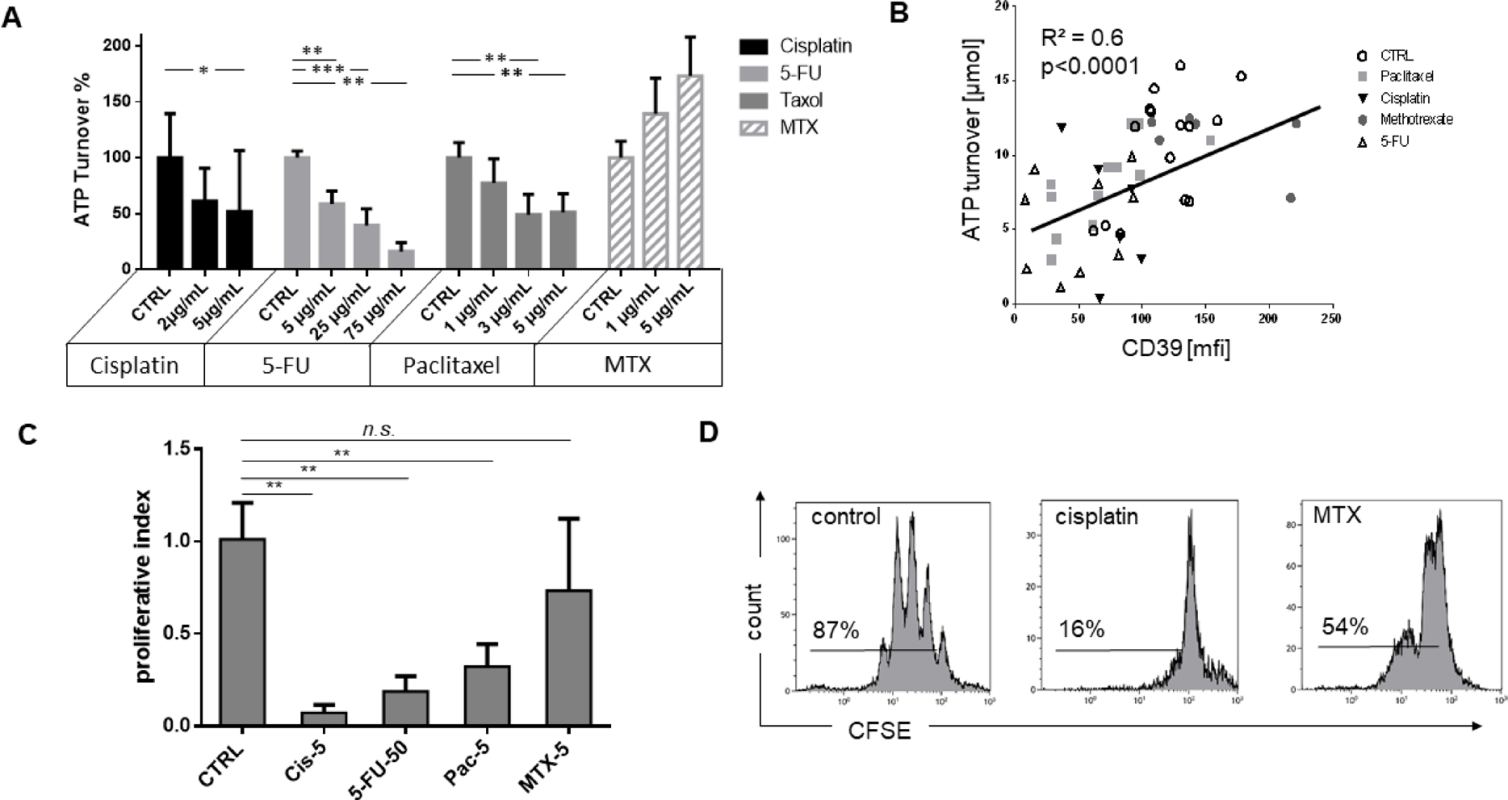
ATP hydrolysis, correlation to CD39 expression, and proliferation of B cells under influence of chemotherapy (**A**) Utilization of exogenous ATP by B cells incubated with 20 µM ATP after 90 minutes detected by luminescence measurement. The data are means ± SD from three or more independent experiments with cells of different HCs each cultured in the presence of one cytostatic drug for 7 days before ATP add-on. Control measurements without treatment are set to 100% on the y-axis. (**B**) Spearman rank correlation of ATP hydrolysis and CD39 surface expression analyzed by flow cytometry. (**C**) Proliferation examined by dilution of CFSE: Left, average of at least three independent experiments, normalized to untreated controls; right, representative examples incubated with 1 µg/mL cisplatin and 10 µg/mL methotrexate. (**C**) Proliferation examined by dilution of CFSE. Average of at least three independent experiments, normalized to untreated controls. (**D**) representative examples incubated with 1 μg/ mL cisplatin and 10 μg/mL methotrexate.

ATP is efficiently hydrolyzed to ADP and AMP by the enzymatic activity of CD39. To quantify the function of this enzyme and further evaluate the influence of chemotherapy, precultured B cells were separated and incubated with one of the cytostatic drugs. The same number of B cells (3 × 10^4^) separated from the cultures were then analyzed in terms of ATP consumption by luminescence assay. Trypan blue dye was used for cell count and to determine viable cells. B cells cultured in the presence of high dose of cisplatin (*p <* 0.05), paclitaxel (*p <* 0.01), and 5-FU (*p <* 0.01) all showed lower ATP hydrolysis than B cells of the same healthy donors cultured in the absence of any cytostatic drug. Furthermore, 5-FU and paclitaxel each induced a significant reduction in ATP consumption, even in low or moderate dosages. In contrast, MTX showed a higher ATP hydrolyzation (*p =* 0.1; Figure 4A). There was a significant correlation between ATP utilization and CD39 expression for all samples (*p <* 0.0001), indicating a direct relationship (Figure 4B). This correlation was found to be particularly significant and dose-dependent in the presence of paclitaxel (*p <* 0.001; Supplementary Figure 1G).

### Adenosine production measured by mass spectrometry

**Figure 5:**
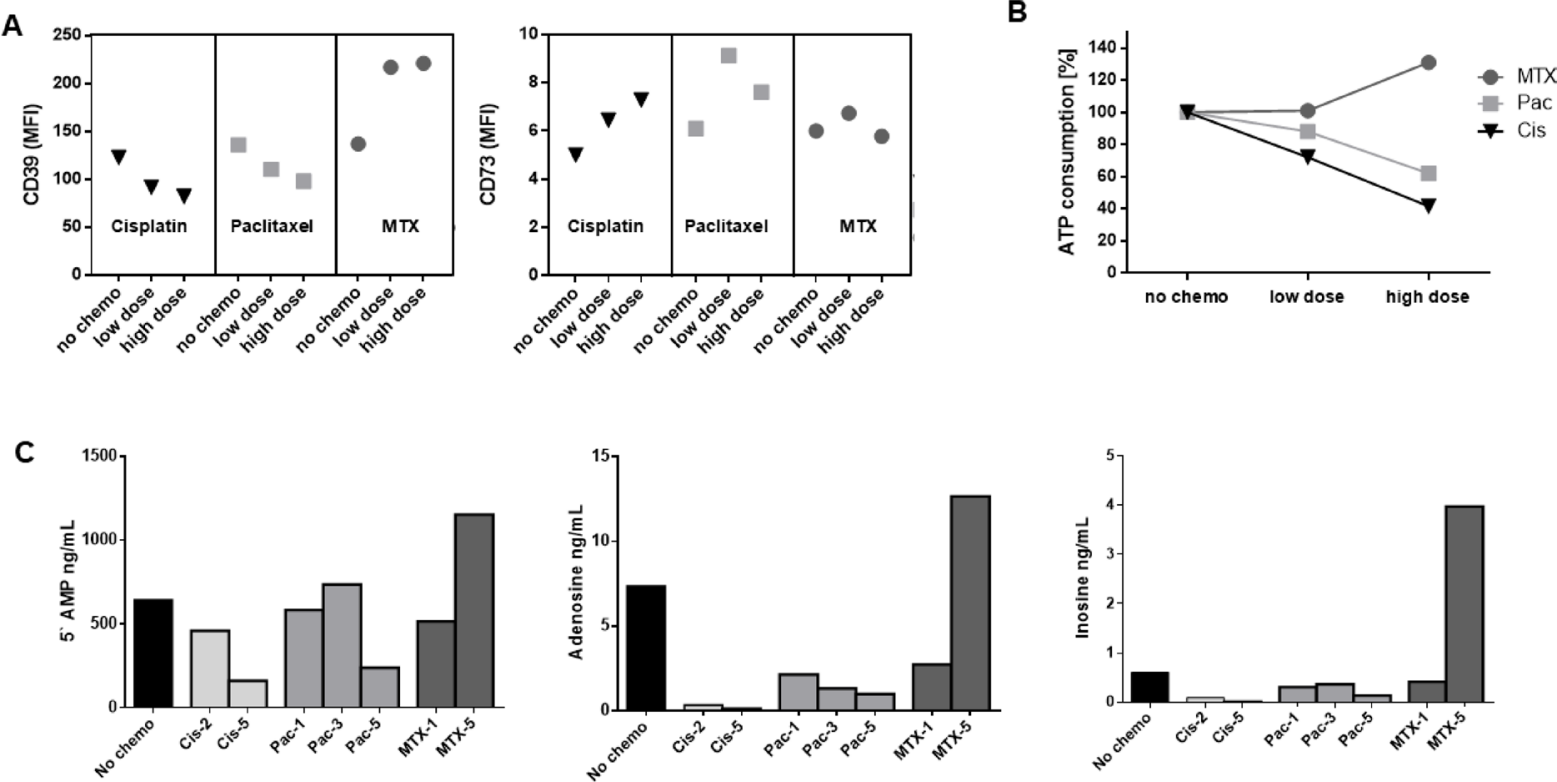
Correlation of B cell phenotype, ATP hydrolysis, and production of its derivates 5′AMP, ADO, and inosine Juxtaposition of B cell treatment from one representative donor to confirm ATP hydrolysis and correlation with the respective phenotype. (**A**) Expression of ectonucleotidases after 7 days of cytostatic treatment as measured by flow cytometry. (**B**) ATP consumption as measured by luminescence. ATP concentrations measured without the influence of chemotherapeutic drugs were set to 100%. (**C**) Production of 5′AMP, adenosine, and derivatives as measured by mass spectrometry. Decreased ATP hydrolysis and adenosine production correlated well with less CD39 expression after treatment with cisplatin and paclitaxel while methotrexate showed opposite effects.

In order to confirm results of ATP hydrolysis measured by luminescence, and therefore, functional characterization of the ectoenzymes CD39 and CD73, ADO production was measured by mass spectrometry. As expected, B cells showing downregulation of CD39 after treatment with cisplatin were less capable of ATP hydrolyzation and produced lower levels of 5′AMP. Due to the lack of substrate, production of ADO and inosine was also reduced. Despite the small number of samples, the data showed a trend towards significance in terms of ADO concentrations (no chemo vs. cisplatin; *p =* 0.07). Figure 5 displays measurements of one representative patient including (A) expression of CD39 and CD73 by flow cytometry, (B) ATP consumption by luminescence, and (C) mass spectrometry results. In accordance with our previous results, levels of 5′-AMP, ADO, and inosine were clearly higher in supernatants of B cell cultures incubated with MTX. While altered CD39 expression influenced consequent ADO production, CD73 varied less and seemed to play a minor role in ADO pathways.

### Drug-dependent suppression of B cell proliferation.

B cells of healthy donors were incubated with cytostatic drugs, and proliferation was measured in CFSE-based proliferation assays as described above. Drugs in concentrations ranging from 1 to 50 µg/mL were added on day 0. All drugs significantly inhibited B cell proliferation except for MTX (Figure 4C, 4D). Cisplatin almost completely blocked B cell proliferation (*p <* 0.001). Paclitaxel decreased B cell proliferation in a dose-dependent manner (Supplementary Figure 2).

### Long-term effects of CRT

**Figure 6:**
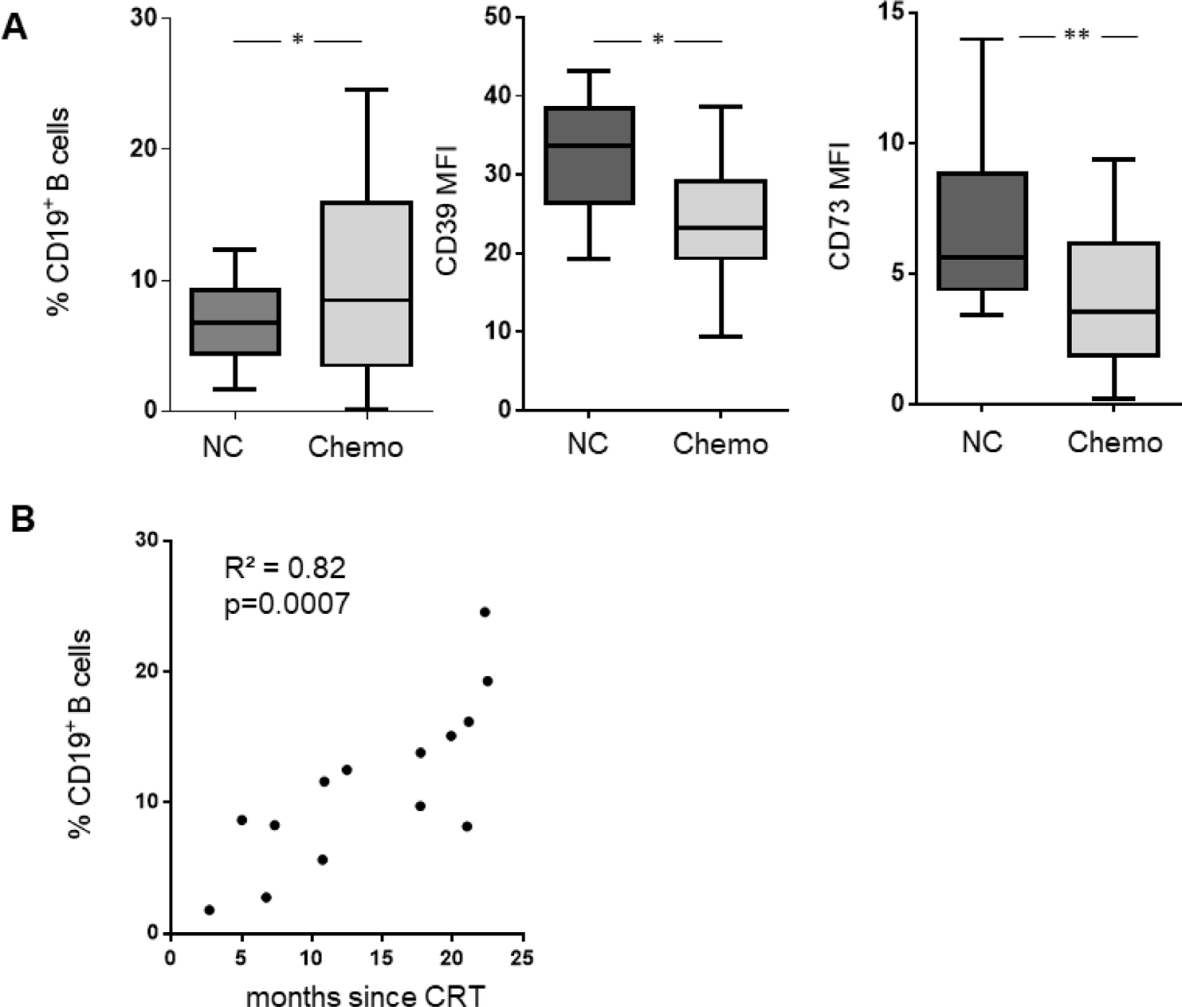
B cell frequency, CD39, and CD73 expression were evaluated in a second cohort of 14 patients with adjuvant CRT and 24 normal controls (**A**) Boxplots show the frequency of B cells before and after CRT. The expression of CD39 and CD73 is decreased after CRT. (**B**) Course of B cell frequency correlated with time after CRT (2–23 months).

Blood samples of patient cohort #2 were collected 14.2 ± 7 months after CRT allowing for interpretation of long-term effects of CRT. The expression of the ectonucleotidases CD39 and CD73 were found to be decreased (*p =* 0.01); Figure 6A). However, the B cell frequency was significantly increased after CRT. In addition, there was a significant correlation between B cell frequency and time-after-treatment (*p <* 0.001) suggesting B cell population recovery after exposure to CRT (Figure 6B).

## DISCUSSION

### Role of B cells in cancer

Effects of systemic cancer treatment on the host immune system are of considerable relevance. The interaction and function of immune cells is affected by any kind of therapy with a consequent reduction of antineoplastic activity. Recently, the role of tumor-infiltrating B cells has been a focus of various research groups and as described above, their impact on tumor growth has been underestimated in the past and is a matter of dispute. In particular, the role of tumor-infiltrating regulatory B cells (TIL-Breg) is not well understood [1]. However, it is reasonable to expect that adenosine-producing B cells have an immunosuppressive effect in the tumor micro-environment.

### Influence of cisplatin on B cells

Here, we show for the first time the effect of systemic chemotherapy on Breg in cancer patients. Platinum-based therapy not only reduces the frequency of Breg but also their ability to produce immunosuppressive adenosine. This effect was demonstrated *in vitro* as well as in two independent cohorts of HNSCC patients. Our previous work has shown opposing effects on regulatory T cells, which are strongly enhanced in frequency and function by platinum-based chemotherapy [33]. It therefore becomes evident that oncologic therapy has different effects on regulatory cell populations in the human immune system. Based on the results of other research groups, tumor escape mechanisms apparently benefit from the presence of immunosuppressive ADO; the dysfunction of the ectonucleotidases CD39 and CD73 is associated with reduced tumor growth [38–40], and up-regulation of CD73 on tumor cells is linked to increased risk for metastasis and increased chemo resistance [41–43]. However, other studies report antitumor effects of ADO [44–46], describing intracellular and extracellular mechanisms leading to apoptosis of cancer cells through ADO.

### Recovery of B cells

In our second cohort of cancer patients, we could show that B cells have a fast recovery rate reaching normal levels about one year after termination of platinum-based chemotherapy. This is in contrast to our previous observations analyzing the Treg (CD4^+^FoxP3^+^) population. There, we demonstrated that the frequency of Treg is increased for up to three years after chemotherapy [33]. Of note, the increased frequency of Treg after chemotherapy is caused by the decreased absolute number of CD4^+^ T cells. There was no increase observed in the absolute number of Treg. These data suggest that although CRT adversely affects both lymphocyte subsets, it has exceptionally detrimental effects on the CD4^+^ T cell population. CD19^+^ B cells seem to be less sensitive in the long-term since they recover faster from CRT than CD4^+^ T cells. From an immunologic perspective, the altered lymphocyte homeostasis may partially explain, why HNSCC cells become resistant to an initially effective therapy.

### Influence of MTX on B cells

When compared with platinum-based chemotherapy, MTX has an opposing effect on the human immune system. In our study population, Breg frequencies increased and showed enhanced ability to produce exogenous adenosine when patients were treated with MTX. These clinical data are supported by our *in vitro* experiments. MTX is not only used as an antineoplastic drug in cancer patients but also as an anti-inflammatory drug, e.g. in rheumatoid arthritis where it is used in a lower dose. Our results show an increased adenosine production in Breg induced by the presence of MTX, whose anti-inflammatory mechanism of action is not fully understood but is thought to be adenosine dependent [47]. It is further postulated by others that caffeine can suppress the therapeutic effect or unwanted side effects of MTX by targeting the adenosine receptors in an anti-inflammatory setting [47, 48]. The importance of the MTX-ADO axis is also confirmed by Tsujimoto *et al.* who found an association between MTX-induced leukencephalopathy and the polymorphism of the ADO receptor A2a in pediatric leukemia patients [49]. Moreover, MTX was demonstrated to suppress the NFkappaB pathway by the release of ADO in Jurkat cells contributing to its anti-inflammatory and antiproliferative effect [50]. Whether these observations can be translated into the antineoplastic effects of MTX in solid cancer patients is yet to be investigated.

Despite the analogy, caution is advised in extrapolating experimental results from arthritis patients to cancer patients as the diseases arise from entirely different conditions. Namely, it is not yet completely understood if immune suppression in the tumor micro-environment of cancer patients is beneficial or not. In addition, great differences may exist in the micro-environment of different kinds of cancer [22]. Interestingly, MTX may even have a promoting effect on adenosine metabolism in cancer cells as shown in glioblastoma cell lines due to the upregulation of the ectonucleotidase CD73 [51].

### Immunotherapy relies on a functional immune system

Aside from the standard regimen of tumor therapy, e.g. surgery, radiation and chemotherapy, a variety of immunotherapeutic approaches have recently found their way into clinical routine. Special potential is seen in the checkpoint modulators, e.g. inhibitors of the programmed death receptor PD1, as they can lead to long-term survival in advanced tumor disease [52]. All immunotherapeutic approaches rely on a functional immune system, and it is not yet clear how to optimally combine the different therapeutic regimens. Modified lymphocyte function and homeostasis induced by CRT could lead to therapy failure. This may be especially true for tumor cells arising from a chronic inflammatory setting, which is the case in HPV^+^ HNSCC. Here, the alteration of extracellular adenosine production may be added to the mechanisms, which result in therapy resistance. On the other hand, these processes may have the potential to overcome CRT failure in an immunosuppressive tumor microenvironment [53]. As recently demonstrated by Maj *et al.*, regulatory cell populations can induce resistance to PD1-mediated therapy in the tumor microenvironment (TME). Due to the oxidative stress in the TME, the preferred mechanism of suppression is adenosine (and not PD-L1, CTLA-4, TGF-b, IL-35, or IL-10) [54]. These findings demonstrate the importance of adenosine in the context of immune suppression and therapeutic failure.

## CONCLUSIONS

Our results add to the understanding of how chemotherapeutic drugs can influence the human immune system and may therefore help to orchestrate standard oncologic therapy with new immune modulating approaches. Giving consideration to the growing importance of tailored cancer treatment, further investigation of the interaction between antitumor immune response and systemic, oncologic therapy may be needed to adapt antineoplastic therapy regimes to individuals.

## MATERIALS AND METHODS

### Patient cohort #1

Peripheral blood samples (*n* = 36) were obtained from 15 HNSCC patients before and after CRT and from 3 patients before and after palliative chemotherapy (11/2013–10/2016). Healthy volunteers (NC, *n* = 20) served as a control group for *in vitro* experiments.

### Patient cohort #2

An additional cohort of HNSCC patients (*n* = 14) donated peripheral blood after CRT, which was terminated 14.2 ± 7 months prior to the respective blood draws. At this time, none of the patients showed evidence of recurrent disease (NED). Blood samples were compared to an additional healthy control group (NC, *n* = 24) in order to analyze long-term effects of CRT. All subjects signed an informed consent form approved by the respective local ethics committee (# 255/14; IRB #991206) according to the declaration of Helsinki. Clinicopathologic and demographic data for both patient cohorts are listed in Tables 1 and 2.

**Table 1:** Patients’ characteristics

	Cohort #1	normal controls	Cohort #2	normal controls
N (female/male)	18 (4/14)	20 (13/7)	14 (1/13)	24 (6/18)
age (range)	59 ± 10 (45–76)	50 ± 12 (19–67)	58 ± 13 (31–86)	28 ± 10 (23–60)
stage				
T1/T2/T3/T4	5/1/6/6		1/4/4/5	
nodal status				
N0/N1/N2	3/3/12		5/2/7	
primary site				
oral cavity	8		7	
pharynx	4		3	
larynx	4		3	
other	2		1	
chemotherapy				
cisplatin	13		12	
carboplatin	1		2	
methotrexate mitomycin	3 1			
primary CRT	8		0	
adjuvant CRT palliative CT	7 3		14 0	

**Table 2:** Patients’ characteristics, cohort #1

#	primary site	stage	sex	Age	chemotherapy	HPV
#1	oral cavity	T3N2cM0	f	49	primary CRT CIS (20 mg/m^2^/d, 2 weeks)	neg.
#2	oral cavity	T3N0M0	m	50	primary CRT CIS (40 mg/m^2^/w, 7 weeks)	n/a
#3	oral cavity	T4aN2cM0	m	61	primary CRT CIS (40 mg/m^2^/w, 7 weeks)	pos.
#4	oral cavity	T1N2bM0	m	68	primary CRT CIS (40 mg/m^2^/w, 7 weeks) CARBO (AUC-2, 3 weeks)	pos.
#5	nasopharynx	T3N2bM0	m	60	primary CRT CIS (20 mg/m^2^/d, 2 weeks)	n/a
#6	larynx	T3N2cM0	f	45	primary CRT CIS (40 mg/m^2^/w, 7 weeks)	n/a
#7	oral cavity	T2N2bM0	m	68	primary CRT CARBO (AUC-6, 2 weeks)	neg.
#8	pharynx	T4aN3bM0	m	76	primary CRT MIT-C (10 mg/m^2^/w, 2 weeks)	n/a
#9	oral cavity	T3N2bM0	m	52	adjuvant CRT CIS (20 mg/m^2^/d, 2 weeks)	neg.
#10	oral cavity	T1N1M0	m	50	adjuvant CRT CIS (20 mg/m^2^/d, 2 weeks) 5-FU (600 mg/m^2^/w, 2 weeks)	neg.
#11	pharynx	T1N2bM0	m	52	adjuvant CRT CIS (20 mg/m^2^/d, 2 weeks)	n/a
#12	larynx	T3N2cM0	m	63	adjuvant CRT CIS (20 mg/m^2^/d, 2 weeks)	n/a
#13	larynx	T4aN1M0	m	53	adjuvant CRT CIS (20 mg/m^2^/d, 2 weeks)	n/a
#14	larynx	T1aN0M0	m	70	adjuvant CRT CIS (30 mg/m^2^/w, 7 weeks)	n/a
#15	acoustic meatus	T4N1M0	f	64	adjuvant CRT CARBO (AUC-6, 2 weeks)	n/a
#16	oral cavity	T1N2bM1	f	46	palliative CT MTX (40 mg/m^2^/w, 6 weeks)	pos.
#17	pharynx	T4aN0M1	m	58	palliative CT MTX (40 mg/m^2^/w, 6 weeks)	neg.
#18	pharynx	T4aN2bM1	m	74	palliative CT MTX (40 mg/m^2^/w, 6 weeks)	neg.

CRT: chemoradiotherapy; CIS: cisplatin (30–40mg/m^2^ weekly for 7 weeks, or 20mg/m^2^ daily in week 1 and 5); CARBO: carboplatin (AUC-area under the curve); MIT-C: mitomycin-C; 5-FU: 5-fluoruracil; MTX: methotrexate. Patients with adjuvant CRT underwent surgery before. Primary treated patients were treatment naive. n/a: not available. #16 was previously treated with CIS/5-FU/cetuximab; #17 and #18 were previously treated with taxane/CIS/5-FU, gemcitabine, cetuximab, and pembrolizumab.

### PBMC collection

Blood samples (50 mL) were drawn into citrate-buffered tubes and centrifuged on Ficoll-Hypaque gradients (Greiner Bio-one, Kremsmuenster, Austria). Peripheral blood mononuclear cells (PBMC) were recovered, washed twice with RPMI medium (RPMI 1640, Gibco, Grand Island, NY) and phosphate-buffered saline (PBS, Gibco), and counted in a trypan blue dye. PBMC from study cohort #1 were cryo-conserved for subsequent analysis. PBMC from cohort #2 were immediately used for *in vitro* experiments.

### FACS antibodies

The following anti-human monoclonal antibodies (mAb) were used for flow cytometry: CD4 AF700 (Cat 56-0048-82), CD73 FITC (Cat 11-0739-42), CD73 PE (Cat 12-0739-42), CD39 PE-Cy7 (Cat 25-0399-42), PD1 (CD279) PE (Cat 12-2799-42), and CCR7 (CD197) PE-Cy7 (Cat 25-1979-42) mAb (all eBioscience, San Diego, CA); CD5 AF700 (Cat 561159), CD19 PE-Cy7 (Cat 555414), and IgM PE (Cat 555783) (all BD Pharmingen, Heidelberg, Germany); CD26 APC (Cat 302710), CD25 FITC (Cat 356105), and CD40 PE-Cy7 (Cat 334322) (all Biolegend, San Diego, CA); CD25 PE (Cat 130-101-426) and IgA APC (Cat 130-093-113) (Milenty Biotec, Bergisch Gladbach, Germany). All mAb were titrated using normal PBMC to establish optimal staining dilutions.

### Surface staining and flow cytometry

Briefly, cells were incubated with mAb specific for surface markers in 50 µl PBS for 30 minutes at room temperature in the dark and washed twice before acquisition for surface marker detection. Flow cytometry was performed using a Gallios™ 10-color flow cytometer equipped with Kaluza^®^ flow cytometry software (both Beckman Coulter, Brea, CA). The acquisition and analysis gates were restricted to the lymphocyte gate based on characteristic properties of the cells in forward and side scatter. At least 10^5^ cells were acquired for analysis. Absolute numbers of CD19^+^ B cells and CD39^+^CD73^+^ Breg were calculated by multiplying their frequency values by the absolute number of lymphocytes obtained from whole blood counts.

### Cell isolation

B cells were separated from PBMC by negative selection using biotinylated anti-CD19 Ab and anti-biotin magnetic beads (B Cell Isolation Kit II, human, #130-091-151, Miltenyi Biotec,) according to the manufacturer’s instruction. The purity of separated cells was always >93% as monitored by flow cytometry.

### Cell culture

Freshly separated CD19^+^ B cells were seeded in flat bottom 96-well plates (10^5^ cells/well) and cultured in RPMI containing 10% fetal bovine serum (PAN Biotech, Aidenbach, Germany), 4 mM L-glutamine, 100 U/mL penicillin, and 100 mg/mL streptomycin (all from Gibco). B cells were stimulated with CD40L (10 ng/mL), hemagglutinin (423 ng/mL, both R&D Systems, Minneapolis, MN), and IL-4 (200 IU/mL, CellGenix, Freiburg, Germany) and cultured for 7 days at 37°C and 5% CO_2_. For *in vitro* testing of chemosensitivity as well as phenotypical and functional effects, the following soluble antineoplastic drugs were added in various concentrations: cisplatin (Teva, Ulm, Germany), methotrexate (Pfizer, Berlin, Germany), 5-FU (Medac, Hamburg, Germany), paclitaxel (Fresenius, Bad Homburg, Germany), and cetuximab (Merck, Darmstadt, Germany). Drug concentrations were in the range of concentrations measured in cancer patients [36]. Cell proliferation was measured by flow cytometry in CFSE-based proliferation assays (Thermo Fisher Scientific, Waltham, MA).

### ATP hydrolysis

CD19^+^ B cells (3 × 10^4^ per well, viable cells counted in a trypan blue dye) obtained from NC, after seven days of chemotherapeutic treatment, were incubated with 20 µM exogenous ATP (Sigma-Aldrich, St. Louis, MO) in 96-well plates for 90 min. The concentration of nonhydrolyzed ATP was determined by measuring the frequency of luminescent events in a luciferase-based detection system (ATPlite Luminescence Assay System, PerkinElmer, Waltham, MA) using a luminescence reader (Infinite^®^ 200 Pro, Tecan, Maennedorf, Switzerland). Control samples included wells with ‘no cells’ or ‘no ATP’.

### Mass spectrometry

B cells from NC were separated by magnetic immunobeads as described previously. For detection of ATP and 5′-AMP hydrolysis, 25,000 cells were incubated in 200 µL PBS in 96-well plates in the presence of 20 µM ATP for various time periods. Control wells contained PBS alone. All experiments were duplicated. Supernatants were collected, centrifuged for 2 minutes at 6,000 × g, boiled for 2 minutes to inactivate ADO-degrading enzymes, and stored at –80°C for subsequent analysis. Purines were measured using liquid chromatography-tandem mass spectrometry by selected reaction monitoring with ^13^C_10_-ADO, ^13^C_10_-5′-AMP, and ^15^N_4_-inosine as internal standards. In this regard, samples were injected into an Acuity ultra-performance liquid chromatographic system (Waters, Milford, MA) and were separated with a C18 column (Waters UPLC BEH C18; 1.7 micron; 2.1 × 100 mm) using the following elution conditions: mobile phase A, 1% acetic acid in H_2_O; mobile phase B, methanol; flow rate, 0.3 mL/min; elution gradient (A/B) was 99.5%/0.5% (0 to 2 minutes), 98%/2% (2 to 3 minutes), 85%/15% (3 to 4 minutes), and 99.5%/0.5% (4 to 5 minutes). Purine levels were analyzed with a TSQ Quantum-Ultra Triple Quadrupole mass spectrometer equipped with a heated electrospray ionization source. The mass spectrometer was operated in the positive ion mode and the following mass-to-charge transitions were monitored: 348→136 for 5′-AMP; 358→141 for ^13^C_10_-5′-AMP; 268→136 for ADO; 278→141 for ^13^C_10_-ADO; 269 → 137 for inosine; and 269 → 137 for ^15^N_4_-inosine.

### Statistics

Statistical analyses were performed using GraphPad Prism Software (GraphPad Software, Inc., La Jolla, CA). Error bars, where displayed, indicate the standard deviation of the mean or median data from replicate experiments. Significance of differences between samples within figures was confirmed using paired or unpaired *t* tests, depending on the experimental setting, with a significant level of α = 0.05. Correlations were calculated by the Spearman test considering *R*^2^ > 0.5 to be significant.

## SUPPLEMENTARY MATERIALS FIGURES


