# Supplementary material for: The influence of chemotherapy on adenosine-producing B cells in patients with head and neck squamous cell carcinoma
Source: Oncotarget. 2017 Dec 20;9(5):5834–47. doi: 10.18632/oncotarget.23533 (PMC5814178; doi:10.18632/oncotarget.23533)
Supplement: Supplementary file 1 [file oncotarget-09-5834-s001.pdf]

The influence of chemotherapy on adenosine-producing B cells in patients with head and neck squamous cell carcinoma

SUPPLEMENTARY MATERIALS

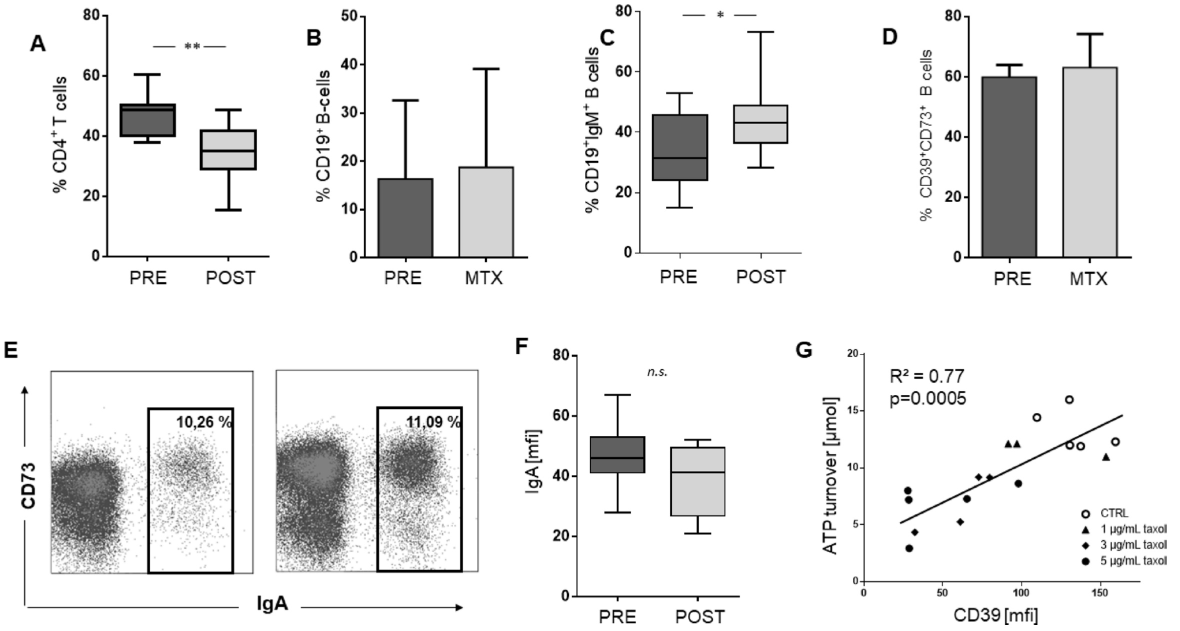

Supplementary Figure 1: (**A** and **B**) Frequencies of CD4$^+$ T cells and CD19$^+$ B cells were determined by flow cytometry before and after a platin or MTX-based therapy, respectively. (**C**) The percentage of IgM-expressing B cells increased after CRT. (**D**) Adenosine-producing B cells showed no alteration in frequency after MTX-based treatment, gated on CD19$^+$ B cells. (**E** and **F**) CD19$^+$IgA$^+$ cells were not affected by systemic antineoplastic treatment. (**G**) CD39 expression on B cells, and therefore ATP hydrolysis, was impaired by paclitaxel dose-dependently.

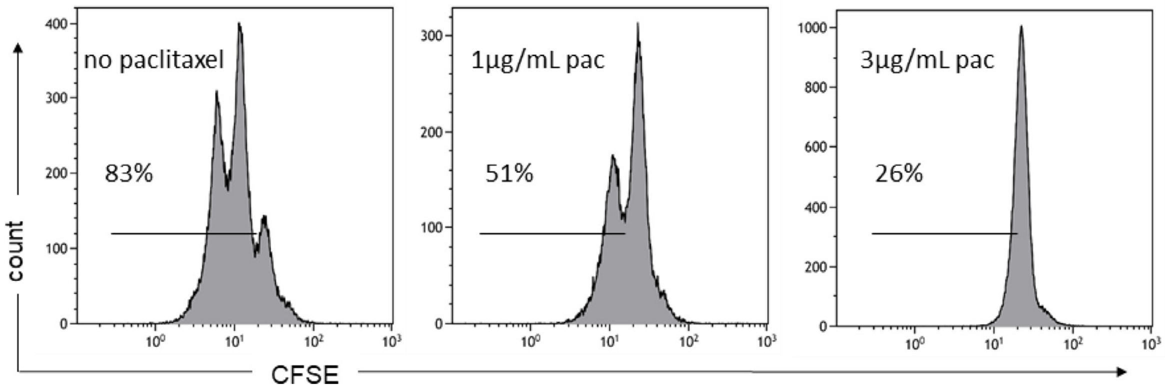

Supplementary Figure 2: Representative example of human B cells (precultured with IL-4, CD40L and hemagglutinin) from one healthy donor incubated with no cytostatic, 1 µg/mL and 3 µg/mL paclitaxel showing dose-dependent impairment of B cell proliferation measured by flow cytometry through dilution of CFSE.
